# Exploring the perceived factors that affect self-medication among nursing students: a qualitative study

**DOI:** 10.1186/s12912-018-0302-2

**Published:** 2018-08-03

**Authors:** Ali Soroush, Alireza Abdi, Bahare Andayeshgar, Afsoon Vahdat, Alireza Khatony

**Affiliations:** 1 Clinical Research Development Center of Imam Reza Hospital, Kermanshah University of Medical Sciences, Kermanshah, Iran; 2 Students Research Committee, School of Nursing and Midwifery, Kermanshah University of Medical Sciences, Kermanshah, Iran; 3 Social Development and Health Promotion Research Center, Kermanshah University of Medical Sciences, Kermanshah, Iran; 4 Nursing Department, School of Nursing and Midwifery, Doolat Abaad, Kermanshah, Iran

**Keywords:** Self-medication, Student, Nurse, Qualitative study

## Abstract

**Background:**

Self-medication is the use of one or more medications without physician’s diagnosis, opinion, or prescription and supervision, which includes the use of herbal or chemical drugs. Todays, self-medication is one of the biggest socio-health and economic problems among nursing students of various societies, including Iran, and because this issue can affected by contextual factors, this study aimed to explore the perceived factors that affect self-medication among nursing students.

**Methods:**

In this qualitative study, a semi-structured interview was conducted with 11 nursing students. The transcript of each interview was reviewed several times and classified into main categories and sub-categories by content analysis. To evaluate this study, Guba and Lincoln’s four criteria, including credibility, transferability, dependability, and confirmability were considered for trustworthiness.

**Results:**

After analyzing the qualitative content of the interviews, four main categories, including educational backgrounds, nature of the disease, access to the media, and beliefs and personal experiences, and ten subcategories, including contact with clinical environment, relative knowledge about medications, simplicity of the disease, recurrence of the disease, influence of the media, use of the internet, believing in own knowledge, positive experiences of traditional medicine, and using own and others’ experiences, were extracted.

**Conclusions:**

It seems that, having a relative awareness about various diseases and medications, which is sometimes associated with taking a few educational courses with an internship, creates a false confidence in student for self-medication and prescribing drugs to others. It would be beneficial if the education system and associated tutors could inform the students about the possible consequences of this issue. By knowing the internal and subjective factors that influence the self-medication, this arbitrary practice can be largely prevented.

## Background

Self-medication is the use of one or more medications without physician’s diagnosis, opinion, or prescription and supervision, which includes the use of herbal or chemical drugs [1]. Today, self-medication is one of the biggest socio-health and economic problems of various societies, including Iran [2]. In some developing countries, many medications are available to public without prescription, thus, self-medication, due to its lower cost, is a replacement for people who cannot afford medical services [3]. This is why in most developing countries more than 60–80% of health problems are associated with self-medication [4]. It is estimated that, about 83% of Iranian people self-medicate [5]. Arbitrary drug administration is common in many societies and is increasing. The prevalence of self-medication in European countries has been reported to be 68%, in USA is 77%, in Kuwait is 92%, in India is 31%, and in Nepal is 59% [6]. On the other hand, drug use pattern is an important indicator in health evaluation. Having adequate knowledge on these patterns helps to identify and determine the prevalence of illnesses, and provides information on how to use health resources [7]. It is also expected that, the well-educated community including university students, is more aware of the danger of self-medication than ordinary people [8]. In this regard, the results of Ehigiator et al. study showed that, most nursing students, midwives and dentists were self-medicating and its influential factors included getting advice from pharmacy staff, friends and other healthcare professionals, as well as previous experiences with the disease [9]. Studies have shown that, the field of study is an underlying factor for self-medication among nursing, midwifery and medical students [10–13]. The simplicity and recurrence of the disease were among other factors of self-medication mentioned in other studies [13–15]. Since most of the factors contributing to self-medication are related to context-based and internal and subjective factors, identifying these factors can help to take interventional measures that are necessary to reduce or eliminate them. As qualitative studies on the perceived factors that affect self-medication in the world are limited, and no qualitative study in this regard has also been conducted in Iran, therefore, this study was conducted to explore the perceived factors of self-medication among nursing students.

## Methods

The present study is a qualitative study with content analysis approach that aimed to explore the perceived factors that affect self-medication among nursing students, which was conducted in 2017. Content analysis is considered as a useful research approach. In this approach, the components or important parts of the content of the existing data are identified and considered [16]. The study population consisted of all nursing students of Kermanshah University of Medical Sciences (KUMS). The samples included nursing students who were self-medicating and had been identified during the previous quantitative study. Hence, in the previous quantitative study entitled: “The prevalence of self-medication among students”, we asked the students to write their phone number and email address in the questionnaire if they are interested to participate in another qualitative study on self-medication. Those students who agreed and had experience of self-medication were recruited for the current study by purposeful sampling.

### Data collection method

Semi-structured interviews were used in this study. The interview questions included: Why do you do self-medication? How and in what cases do you do self-medication? What are the perceived factors that affect self-medication? In order to clarify the concepts, probing questions were also asked, including “Please explain more”, why and how?

After obtaining the approval of Ethics Committee of Kermanshah University of Medical Sciences (Kums.rec.1396.25), and explaining the aims of study to the participants and obtaining written consent to record their voices, interviews were conducted at the convenient place agreed by the researcher and the participants. Every interview was recorded by a tape recorder. Each interview lasted between 30 and 50 min. After each interview, the recorded interview content was carefully reviewed several times, and then typed verbatim. This was done to increase the accuracy of the information transferred to paper and to further control the information.

### Data analysis

Data analysis was done simultaneously with data collection. The data resulted from the interviews were analyzed simultaneously using qualitative content analysis. In content analysis, information units are identified in response to each question. These units, which present as codes, may include concepts, phrases or words that are clearly, understandably and systematically classified in different categories according to the content and, on the basis of their theoretical significance [17].

Among the Mayring’s processes of qualitative content analysis, two main approaches are considered for the expansion and classification of appropriate text components, which include inductive and deductive methods. Inductive content analysis is a process that is used to extract classes or themes from raw data based on valid conclusion and interpretation. Deductive content analysis is used when the analysis structure is operational based on previous knowledge and the purpose of study is to examine the theory [18]. Inductive content analysis method was used in the present study, because there was little information on the perception of nursing students about self-medication. The transcripts of each interview were reviewed several times and a general understanding of the participants’ statements was obtained. Then, the semantic units were shaped and initial codes were extracted. The codes that were conceptually similar were categorized into a group, thus categories and subcategories were determined. The data saturation was reached at the eighth interview, but further interviews were conducted with three other students to ensure the saturation. The data saturation criteria vary according to sample variety, participant selection method, data collection method, budget, and available resources, but saturation of data occurs when no new code is obtained during data collection and analysis, according to at least two researchers specializing in qualitative research [19].

### Trustworthiness

Guba and Lincoln have proposed four criteria for the evaluation of interpretive research, including credibility, transferability, dependability and confirmability [20].

A. **Credibility:** The credibility of a study is the degree to which the findings of the study are true, and reflect the purpose of the research and social reality of the participants [16]. In this study, to enhance the credibility of the qualitative content analysis, methods such as continuous and long-term engagement with samples, control of interpretations against raw data, peer review, and review of the participants were used.
B. **Transferability:** The main goal of qualitative research is not to generalize the findings, as in quantitative research. It is not the duty of researcher to provide guidance for generalizability, but the researcher is required to provide a set of data and a description that is sufficiently rich for other researchers, to enables them to make informed decision about the generalization of the findings in other fields and conditions [18]. In this study, the perceived factors that affect self-medication among nursing students were examined carefully and all cultural and background characteristics of the samples were explained. Finally, findings of the study were given to three nursing students, beyond our participants, who have been self-medicating, and their personal experiences were compared with the results of the study; thus the students’ experiences were in line with our participants’ results, which confirm transferability.
C. **Dependability:** Dependability indicates the degree to which the researcher has been non-biased and to what extent the findings of the study are in accordance with the responses of the participants and not influenced by the researcher’s bias or interests. In this study, all stages of the study were described step-by-step to be properly judged during the external audit.
D. **Confirmability:** The confirmability is the degree of agreement between several independent individuals about the accuracy and relevance of the meanings of the data. In this regard, some of the interviews and transcripts, along with coding, were provided to two other research colleagues who were specialized in qualitative research and confirmed the accuracy of coding.

To manage the data, Maxqda software version 10 was used.

## Results

**Fig. 1:**
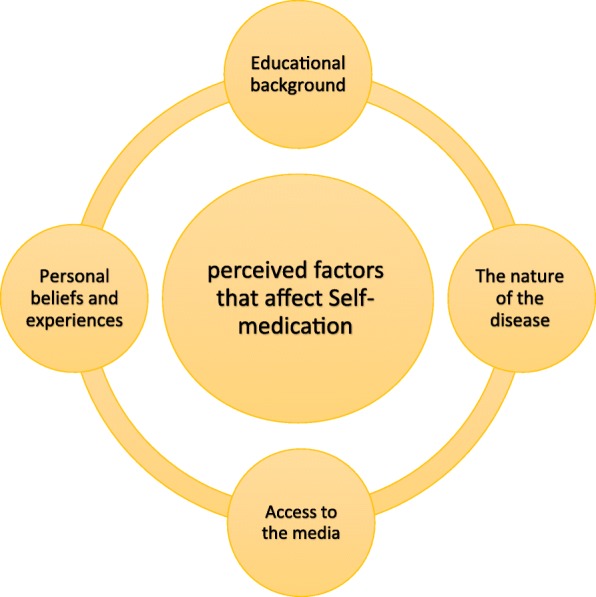
Perceived factors that affect self-medication among nursing students

In this study, 11 nursing students with the age of 21–34 years old were enrolled, including 4 males and 7 females from whom, six were graduate students and five were undergraduate (Table 1). After analyzing the qualitative content of the interviews, four main categories and nine subcategories were extracted. The main categories included “educational background”, “nature of the illness”, “access to the media” and “personal beliefs and experiences”, which also contained 10 subcategories (Table 2) (Fig. 1).

**Table 1:** Participants’ characteristics

Participants	Age range(yrs.)	Grade
First	30–35	MSc.
Second	20–25	BSc.
Third	25–30	MSc.
Fourth	20–25	MSc.
Fifth	20–25	MSc.
Sixth	25–30	MSc.
Seventh	25–30	BSc.
Eighth	25–30	BSc.
Ninth	25–30	BSc.
Tenth	20–25	BSc.
Eleventh	30–35	MSc.

**Table 2:** Classes and sub-classes

Classes	Sub-Classes
1. Educational background	1. Contact with clinical environment 2. Relative knowledge about drugs
2. The nature of the disease	1. Simplicity of the disease 2. Recurrence of the disease
3. Access to the media	1. Use of the Internet 2. Influence of the media
4. Personal beliefs and experiences	1. Believing in own knowledge 2. Positive experiences of traditional medicine 3. Using own and others’ experiences

### Educational background

During the interview process, all students stated that, the educational background could lead to self-medication. In this regard, two sub-categories, including “contact with clinical environment” and “relative knowledge about drugs” were formed, which are subsequently explained.

#### Contact with clinical environment

Students with a history of nursing work in a clinical setting believed that, working in the clinic and with patients, as well as being aware of prescribed drugs for various diseases, could help them to treat non-complicated diseases. They stated that, they have adequate ability to treat simple illnesses. In the first interview, a 34-year-old woman who was an MSc nursing student and had ten years of experience at the clinical settings stated: “Considering the work experience and the knowledge that I have about drugs and diseases ... I try to treat simple illnesses myself”. In another interview, another 27-year-old female student pointed out:“ For example, in cases where I feel that, the disease is similar to the previous illness I have seen at my workplace, I use the same prescription”. (Eighth Interview, a 27 years old female MSc nursing student).

Some students also referred to the access to experienced physicians and colleagues as factor for self-medication. They indicated that, this issue is an obstacle to physician visit for non-complicated illnesses. Moreover, asking the physicians to prescribe medication in the health insurance booklet and physicians’ acceptance was another perceived factor for self-medication. In this regard, a nursing student said: “As I work in the ward, I ask the physicians to write and prescribe medication in my insurance booklet and stamp it, or I write medication myself and ask the doctor to stamp it.” (First interview, a 34 years old, female postgraduate student).

#### Relative knowledge about drugs

All participants believed that, they were familiar with many medications because of their pharmacological modules, and some participants believed that, they were somewhat familiar with the complications of the drugs they were taking and could use this information for self-medication. Also, two postgraduate students stated that, the expectation of others, because of their field of study, could be another perceived factor for self-medication and giving medication advice to others.

In the ninth interview, a student stated that “passing a pharmacology module is very effective in self-medication, for example, I used propranolol tablets to reduce the anxiety. During my bachelor’s degree study, I began self-medicating as soon as I became aware of it, and I am still continuing it; I also know its complications”. (Ninth interview, a 27 years old, male MSc student).

Another student stated that, she uses simpler medications to prevent side effects: “For example, someone who uses antibiotics that is not suitable for his/her infection due to ignorance, in addition to developing resistant to antibiotics, he/she will not get any better and when it comes to a bigger problem, antibiotic is no longer effective”. “I use simpler and less risky drugs when I self-medicate, and I do not buy a dangerous drug from the pharmacy.” (Third interview, a 27 years old, female MSc student).

One student, in regard to her own and others’ expectation stated: “For example, when I feel that I have a problem and I need to go to doctor, people say that, you are a nurse and do not need to go to doctor. You want to go to doctor to say what. You know, they induce that, because you study and work in this field, you must be aware of such knowledge and it is not good if you to say you don’t know. I sometimes have to give medical advice to others unwillingly, as I don’t want to put myself under the question, or stop people saying that, she is a nurse but doesn’t know anything, or she ignores us”. (Eighth interview, a 27 year old, female MSc student).

### The nature of the disease

Another perceived factor for self-medication that was suggested by the participants was the nature of the disease. This category consisted of two sub-categories, including “simplicity of the disease” and “recurrence of the disease”.

#### Simplicity of the disease

Participants stated that, they self-medicate because of the type of disease, and do not visit physician for simple illnesses, for example for illnesses such as common cold. A 27-year-old MSc nursing student believed that in some cases, the illness is not that serious to require doctor visit, and said: “For simple illnesses like headaches and stomachache I take drug .... Sometimes someone says the illness is not serious, for example, why should I go to doctor for constipation. It is both laziness, and you think that this is a simple illness and does not require a doctor visit”. Another student stated that, he would self-medicate for non-complex diseases and would prefer prevention to treatment: “It depends on the type of disease, if it is simple, I can do something about it, but if it is diabetes for example, I cannot. I think prevention is much better than treatment ... In minor cases, such as a cold or a toothache, I know what to use.”(Fourth interview, a 21 years old, male undergraduate student).

#### Recurrence of the disease

Most students believed that, they did not need to spend time and money on their recurring illness because of their previous knowledge about the treatment, and they could self-medicate. They believed that, if they go to the doctor, they will get the same prescription. In this regard, an undergraduate student stated: “If it is a routine problem that has happened ten times before, and the same medications are being prescribed in every visit, this leads to self-medication.” (Seventh interview, a 28 years old, female undergraduate student).

Another student said: “Well, I have had the same problem in the past and the doctor wrote certain drugs for me. So, I do not pay any money, nor I pay MRI or CT scan money, this repetition makes you feel that you know what to do, and what to use and recommend to others.” (Eighth interview, a 27 years old, female MSc student).

### Access to the media

Most students, who were self-medicating, used internet for information on medications and considered it as the source of information. Also, some of them considered the media, especially national television, to be effective in self-medication.

#### Use of the internet

Participants considered the availability and affordability of the internet as a reason for self-medication, although some students believed that most websites were unreliable. In this regard, an undergraduate student stated: “The internet can be a source of information, but not all websites ... If I want to do some search, I’ll go to Latin and up to date websites, and by studying them, I will be a head and neck above my friends and even university tutors.” (Tenth interview, a 21 year old, female undergraduate student).

Another student also said: “I use the internet and pharmacology books for self-medication, but I use internet more often. Although, sometimes I see there are many contradictory information sources, I try to consider a summary of them.”(Fifth interview, a 22 years old, male undergraduate student).

#### Influence of the media

One of the culture-building practices in any field is the use of public media. In regard to self-medication, the media is certainly an influential factor, and can be used properly with control and planning. Some nursing students stated that, giving information about medication and treatment through the media may leads to blind self-medication in some people. In the seventh interview, a 28-year-old female undergraduate student stated: “The programs in which a doctor is talking on television are often harmful, because ordinary people do not properly understand the message, or they take some parts of the message they like and think they know everything about that disease, so they prescribe medication for themselves and others.”

### Personal beliefs and experiences

Nursing students are influenced by their field of study, work environment, beliefs and experiences that lead to self-medication. Surely, part of these beliefs and experiences come from the society, and many ordinary people in the community also believe them. This category consisted of 3 subcategories, including “believing in own knowledge”, “positive experiences of traditional medicine,” and “using own and others’ experiences”.

#### Believing in own knowledge

Participants believed that, their knowledge was at such level that they could treat themselves in simple diseases and do not need to go to doctor. In this regard, a postgraduate student stated: “At times, I think doctors do not know more than us, and I do not really trust them. Because when I look at their prescription, sometimes I see it is wrong and I know how to correct that mistake, but since I cannot prescribe drugs, my hands are tied. However, when it comes to my own case, I can prescribe drugs for myself.” (Ninth interview, a 27 years old, male MSc student).

#### Positive experiences of traditional medicine

Some students felt that herbal remedies are a good substitute for some chemical drugs because of their low side effects. Part of this issue also seemed to be related to the advice of elderly for using herbal medicines. Also, the positive experiences of peers in the use of herbal medicines were effective in the students’ self-medication.

In this regard, one student said: “Self-medication is more likely to go under the complementary medicine, because people think it does not really have any negative consequences. Therefore, they use complementary medicine and their friends and relatives also advise them to take herbal remedy and say; take that herbal remedy and you’ll be fine ... I mean, I’d rather mix honey and tea and drink it than take diphenhydramine syrup.” (Ninth interview, a 27 years old, male MSc student).

#### Using own and others’ experiences

Some students stated that, the use of medications and knowing about their complications and side effects helped them to self-medicate in some cases. Also, in cases where they have been satisfied with the use of a drug to treat a disease, and were aware of its relative safety, they have been advising it to others and close people.

In the fifth interview, a 22-year-old male student who was doing bachelor’s degree in nursing, in regard to others’ experiences in similar diseases, stated: “I was trying to get help from those who had similar disease and had gone to visit a doctor for it. I think everyone is doing it and self-medicating.”

A 27-year-old female MSc nursing student talked about testing and drug errors and believed that, after using a drug, she could notice its effect, and if it was effective, continued to use it. In this regard she stated: “I did not know there were other medications that are good for headaches before I become nursing student. I took them couples of times and then I realized they are good. It was like trial and error. I had migraine, so I tried ergotamine and I became well.”

## Discussion

The purpose of this study was to explore the perceived factors of self-medication among nursing students. The results showed that, one of the factors contributing to self-medication was educational background from the perspective of nursing students, so that having contact with the clinical environment and having relative knowledge of diseases was contributing to this behavior of the nursing students. Evidence suggests that, the work environment, getting advice from colleagues and having pharmacological knowledge are effective in self-medication of medical science students [9, 12–14]. For people who have sufficient knowledge about self-medication in simple diseases, the self-medication in addition to being safe it could also be beneficial. According to the World Health Organization (WHO), self-medication is part of the self-care process and can reduce the pressure on the health system and lead to optimal use of facilities, especially in low-income health care settings [21, 22].

The nature of the disease was another cause of self-medication among nursing students. The simplicity and recurrence of illnesses were important factors for self-medication among nursing students. Evidence suggests that, the highest level of self-medication among medical science students is related to simplicity of the diseases, and one of the effective factors in self-medication in these students is the insignificance and non-complexity of the diseases [11, 13–15, 21, 23–25]. From nursing students’ point of view, simple illnesses such as colds and headaches can be cured by relying on previous knowledge and experiences. In our opinion, self-medication can be dangerous even for simple illnesses, if is done with insufficient knowledge.

Another perceived factor behind self-medication was the access to media and internet. In a study, 17.6% of nursing students referred to the internet as one of the reasons for self-medication [13]. Results of another study indicated that, paper and electronic media advertisement is effective in the self-medication of students [26]. Medical information on the internet is incomplete and often invalid, which can be dangerous. Among the potential dangers arising from information available on the internet and the mass media, we can refer to false self-belief in illness, false diagnosis, and false self-confidence.

Beliefs and experiences were another cause of self-medication in nursing students. This category consisted of several subcategories including; believing in own knowledge, own and others’ previous experiences, and positive experiences of traditional medicine. The results of a study indicated that, medical students intend to have an active role in their health care due to their high level of trust in their own knowledge [12]. In another study, one of the causes of self-medication in medical students was having confidence in own knowledge [14]. Due to having a pharmacological module and attending clinical setting, medical students have an incomplete knowledge that enhances their confidence in the diagnosis and treatment of the diseases, which can endanger their health.

Another perceived factor of self-medication was the use of own and others’ experiences. In this regard, the results of a study showed that, previous history of personal use of medications and counseling was one of the main factors behind self-medication in medical students [15]. In another study, 38% of medical students used the experiences of the elderly and classmates as sources of information on self-medication, and 63.6% of them prescribed drug to others, especially family members, friends and peers [25]. Results of another study showed that, more than half of the medical students used their old prescription to treat the same illness [13]. Using others’ experiences can be highly hazardous and lead to exacerbation of the disease and drug resistance, as individuals may have inadequate knowledge about drugs and their complications. Symptoms and illnesses may also be similar and differential diagnosis of them is only possible by a valid physician.

We found that, some nursing students used herbal medicines for self-medication, because of the relatively low side effects of herbal drugs compared to chemical drugs. Evidence suggests that, students also use herbal medicines for self-medication in other countries [7, 27–30]. In our opinion, the use of herbal medicines with knowledge and awareness can be beneficial otherwise it may cause serious risks. Traditional medicine can be used to deal with such problems.

One of the limitations in this study was the limitation of the generalizability of the findings, which is the nature of the qualitative research. Our study was conducted on nursing students, so due to the high prevalence of self-medication among different medical science students, it is suggested to assess and compare the perceived factors of self-medication among students of different medical science disciplines.

## Conclusions

The factors of self-medication included the educational backgrounds, nature of the disease, access to the media, and personal beliefs and experiences. It seems that, having relative awareness about various illnesses and medications, which is sometimes associated with passing a few lessons and modules with internship, creates a false trust in the student for self-medication and prescribing drugs to others. It would be beneficial if the consequences of this problem could be taught to students by the education system and related university tutors. By knowing the internal and subjective perceived factors that affect self-medication, we can largely prevent this arbitrarily practice.
